# Misdiagnosis of sphincter of Oddi disorder treated as familial Mediterranean fever for ten years: A case report

**DOI:** 10.1016/j.amsu.2022.103295

**Published:** 2022-01-25

**Authors:** Mohamad Shadi Alkarrash, Mohammad Nour Shashaa, Roaa Rhayim, Ziad Aljarad

**Affiliations:** a Faculty of Medicine, University of Aleppo, Aleppo, Syria; b PhD Gastroenterology Department, Aleppo University Hospital, Aleppo, Syria

**Keywords:** Misdiagnosis, Sphincter of oddi disorder, Familial Mediterranean fever, Abdominal pain, Medical errors, FNF, familial Mediterranean fever, SOD, Sphincter of Oddi dysfunction, CBC, complete blood count, WBC, white blood cell, INR, internal normalized ratio, AST, aspartate aminotransferase, ALT, alanine aminotransferase, HCV, hepatitis c virus, EGD, Esophagogastroduodenoscopy, HP, Helicobacter Pylori, CT, computed tomography, CBD, common bile duct, MRI, magnetic resonance imaging, MRCP, magnetic resonance cholangiopancreatography, ERCP, endoscopic retrograde cholangiopancreatography, NSAID, nonsteroidal anti-inflammatory drug, DXA, dual-energy X-ray absorptiometry, IBS, inflammatory bowel syndrome

## Abstract

**Introduction:**

Sphincter of Oddi dysfunction is a rare disease caused by sphincter of Oddi functional or mechanical abnormality. Misdiagnosis of familial Mediterranean fever is very high due to overlapping symptoms with many diseases. Our case is the first case report in the medical literature which describes the misdiagnosis of Sphincter of Oddi dysfunction as familial Mediterranean fever.

**Case presentation:**

A 46-year-old woman presented with recurrent episodes of abdominal pain and arthralgia. The patient had familial Mediterranean fever for ten years which was diagnosed clinically without performing genetic tests. Analysis of the mutation in the MEFV gene was performed and was negative. Thereby, the diagnosis of familial Mediterranean fever was eliminated and colchisine was discontinued. Afterward, laboratory and radiological tests were performed, and the diagnosis of sphincter of Oddi disfunction was confirmed. The patient underwent biliary sphincterotomy and take sulpiride daily.

**Discussion:**

The most common diseases were misdiagnosed with familial Mediterranean fever are appendicitis, acute rheumatic fever, gastrointestinal diseases and inflammatory arthritis. Endoscopic retrograde cholangiopancreatography with Manometry of the Sphincter of Oddi is the gold-standard test.

**Conclusion:**

Sphincter of Oddi dysfunction may interfere with many other disorders and should be considered as a differential diagnosis for any recurrent abdominal pain. Misdiagnosis of familial Mediterranean fever is common in endemic countries due to the reliance on clinical symptoms without analysis of the mutations in the MEFV genes particularly, before 1997.

## Highlights

• SOD should be considered as a differential diagnosis for any recurrent abdominal pain.
• Misdiagnosis of familial Mediterranean fever is common in endemic countries.
• ERCP with Manometry of the Sphincter of Oddi is the gold-standard test.

## 1 Introduction

Medical errors are widespread worldwide, even in developed countries and have an important part in medical practice.

According to the Harvard study, the medical errors were 14% in total; medication errors were the most common type (19%) following by wound infections (14%), technical complications (13%) and diagnostic mishaps (8%) [1].

The proportion of medical errors due to neglect was the highest for the diagnostic mishaps (75%) and 6% from its were due to physician practicing outside of the expertise area [1].

In the USA, medicolegal litigation due to misdiagnosis (26%) emulates surgical accidents (25%) [2].

Misdiagnosis of familial Mediterranean fever (FMF) is very high due to overlapping symptoms with many diseases.

Particularly with appendicitis, acute rheumatic fever and gastrointestinal diseases [3].

Sphincter of Oddi dysfunction (SOD) is a rare disease caused by sphincter of Oddi functional or mechanical abnormality [4] and there are more than 778 articles on sphincter of Oddi dysfunction have been published in the English medical literature [5].

The prevalence of SOD is 1.5% in the general population and may occur at any age but is most commonly encountered in females aged 20–50 years [6].

SOD presents with recurrent biliary pain with or without hepatic enzyme elevation and may be accompanied by nausea, vomiting, and eructation [4]. This symptoms are often overlapped with many diseases (pancreatic and biliary diseases) and the risk of misdiagnosis is very high. Therefore, the disease should be considered as a differential diagnosis in any patient with recurrent abdominal pain. Treatment include noninvasive and invasive interventions. ERCP with sphincterotomy is often highly effective and generally safe for patients with SOD. We report the first case report in the medical literature which describes the misdiagnosis of Sphincter of Oddi dysfunction as familial Mediterranean fever. This case is reported accord to the SCARE criteria [7].

## 2 Case presentation

**Fig. 1:**
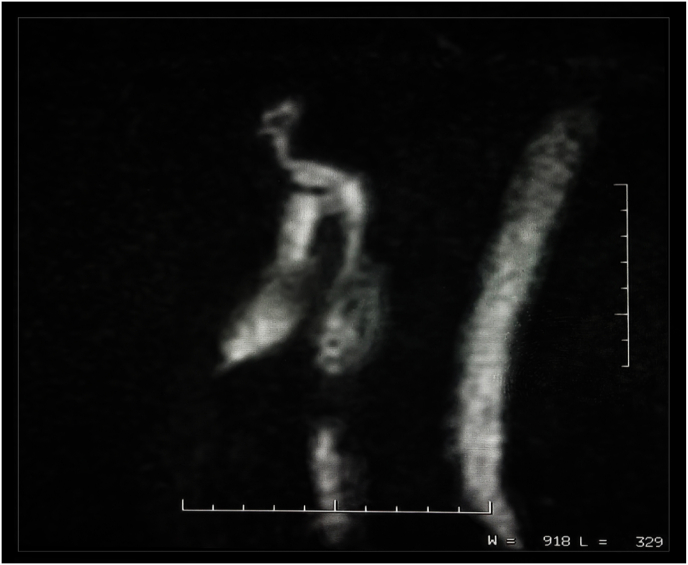
A magnetic resonance cholangiopancreatography (MRCP) image reveals no abnormalities and no obstructive in the biliary ducts.

A 46-year-old woman presented to the clinic with recurrent episodes of upper abdominal pain including the epigastric and right hypochondriac areas, with radiation sometimes to the right shoulder. The patient had generalized arthralgia and eructation for several hours but no nausea or vomiting. She did not use alcohol and quit smoking a year ago. The patient was diagnosed with familial Mediterranean fever (FMF) for ten years, which was diagnosed clinically because the genetic tests were not available and she took colchicine without important symptoms improvement. She also underwent an oophorectomy due to ovarian cyst and hysterectomy due to endometrial polyp. Moreover, the patient had an allergy to penicillin. Physical examination showed localized tenderness in the right hypochondriac area and a palpable liver swelling. Murphy's sign was positive, but no splenomegaly, guarding and jaundice were found. Her vital signs were within normal limits. Straight away, analysis of the mutation in the MEFV gene was performed and was negative. Thereby, the diagnosis of familial Mediterranean fever was eliminated and colchisine was discontinued. Afterward, laboratory tests including hemoglobin - Complete blood count (CBC) - white blood cell (WBC) - glucose - blood urea - serum creatinine - internal normalized ratio (INR) - total serum bilirubin and both conjugated and unconjugated bilirubin - Aspartate aminotransferase (AST) - Alanine aminotransferase (ALT) all were within normal. Anti-HCV antibodies and hepatitis B surface antigen were negative, and C- reactive protein was 95 mg (high). Esophagogastroduodenoscopy (EGD) findings revealed inflammatory features, Helicobacter Pylori (HP) was treated with triple therapy combination. Inspite of treatment was completed the patient was still suffering. Thus, abdominal computed tomography scan (CT) showed no abnormalities and abdominal ultrasound showed semi contracted gallbladder without wall thickening or calculi and a dilated common bile duct (CBD), so an obstructive disorder was Suspected. The abdominal magnetic resonance imaging (MRI) and magnetic resonance cholangiopancreatography (MRCP) were performed to rule out the presence of obstructive causes and showed no abnormalities [Fig. 1]. The patient was anxious and the previous findings are compatible with the criteria of Rome III for Sphincter of Oddi disorder (SOD). Subsequently, the diagnosis of SOD was highly suggested. Afterward, the patient underwent endoscopic retrograde cholangiopancreatography (ERCP) which showed a severe contractile of the sphincter of Oddi and biliary sphincterotomy was performed immediately because the Manometry of the SO is not available in Syria. Depending on the previous clinical and radiographic findings, the diagnosis of sphincter of Oddi disorder was confirmed and according to Milwaukee classification for biliary SOD, the patient had (SOD) type 2. Consequently, the patient was given sulpiride 50 mg daily, nonsteroidal anti-inflammatory drug (NSAID) during the episodes and the patient was recommended to have a fat free diet. Because of generalized arthralgia in addition to the previous oophorectomy, a Dual-energy X-ray absorptiometry (DXA) scan was performed and showed evidence of osteoporosis due to T scores of −3.2 at the lumbar spine and −2.3 at the femoral neck. Consequently, alendronate sodium trihydrate 70 mg, vitamin D supplements and Calcium compounds were given. The patient's repeating follow-up revealed a lack in the recurrence of the episodes but not resolved completely.

## 3 Discussion

Sphincter of Oddi dysfunction is a rare clinical syndrome caused by sphincter of Oddi spasm, stricture, or inappropriate relaxation [8]. SOD is a rare disease in the literature that is currently highly misdiagnosed because its clinical manifestations can mimic other etiologies and it is not considered by a lot of clinicians as a differential diagnosis in any patient with recurrent abdominal pain. Consequently, it can easily be confused with other disorders such as FMF, especially in cases when the patient in an epidemic area presents with recurrent episodes of fever, abdominal and joint pains, and gastrointestinal problems without other suggestive findings for a specific disorder Such our patient who had abdominal pain due to SOD and osteoporosis was the underlying cause for her arthralgia. Analysis of the mutations in the MEFV genes differentiates between these two conditions. Our case is the first case report in the medical literature which describes the misdiagnosis of Sphincter of Oddi dysfunction as familial Mediterranean fever. SOD is associated with abdominal pain in the epigastrium and right upper quadrant that lasts around 30 minutes to several hours and is accompanied by nausea and vomiting. The elevation of liver or pancreatic enzymes, common bile duct dilation can also be found [9]. Multiple potential risk factors of SOD include female gender, hypothyroidism, inflammatory bowel syndrome (IBS), prior pancreatitis, cholelithiasis, alcohol use disorders and exogenous medications [8,10].

Hence, diagnosis of SOD is a clinical challenge; endoscopic ultrasound, computer tomography (CT), and magnetic resonance cholangiopancreatography (MRCP) are non-specific for SOD but can rule out other pathology [9]. Endoscopic retrograde cholangiopancreatography (ERCP) with Manometry of the SO is the gold-standard test; basal pressures ≥40 mmHg indicate SOD [11]. Manometry requires a professional Gastroenterologist with special equipment that is not available at the majority of institutions and in 13–40% of patients with type 1 SOD, the Manometry was considered not a confirmatory test [12]; what is more, it is associated with up to 30% increased risk of iatrogenic pancreatitis [13]. Laboratory evaluation (liver function test, amylase, and lipase) are not specific but should be performed to determine SOD types. Milwaukee classification classifies SOD into types 1, 2, and 3 according to clinical presentations in addition to the laboratory and imaging abnormalities [14]. The options for treatment of SOD include noninvasive and invasive interventions; noninvasive therapies include calcium channel blockers, tricyclic antidepressants, glyceryl trinitrate and somatostatin; conservative treatment is preferred for the patients with type III SOD [9]. Combinations of multiple medications showed better levels of symptom improvement [15]. Invasive intervention is ERCP with sphincterotomy which often highly effective and generally safe for patients with SOD. Hence, patients with type I and type II sphincter of Oddi dysfunction should be referred for management with sphincterotomy due to reports of 90% and 70% resolution of symptoms in types I and II SOD, respectively [8]. The most common diseases were misdiagnosed with FMF are appendicitis, acute rheumatic fever, gastrointestinal diseases (like in our case) and inflammatory arthritis [3]. This misdiagnosis made the patient use colchicine for a long time which made her at high risk for side effects such diarrhea, rhabdomyolysis and neuromyopathy in addition to myelosuppression [16].

## 4 Conclusion

SOD symptoms could interfere with many other disorders and should be considered as a differential diagnosis for any recurrent abdominal pain. The diagnosis of SOD is a clinical challenge and requires the exclusion of other diseases before confirming the diagnosis. We recommend for all patients who were diagnosed clinically with FMF previously to be reevaluated by analysis of the mutation in the MEFV gene because genetic testing was not available before 1997.

## Ethical approval

Not required for case reports at our hospital. Single case reports are exempt from ethical approval in our institution.

## Sources of funding

There are no funding sources.

## Author contribution

MSA and MNS: wrote the manuscript. RR: data collection, designed the manuscript and corresponding author. ZA: managed the patient, edited and supervised the manuscript. All authors read and approved the final manuscript.

## Guarantor

The Guarantor is the one or more people who accept full responsibility for the work and/or the conduct of the study, had access to the data, and controlled the decision to publishMohammad Nour Shashaa.

## Consent

Written informed consent was obtained from the patient for publication of this case report and accompanying images. A copy of the written consent is available for review by the Editor-in-Chief of this journal on request.

## Provenance and peer review

Not commissioned, externally peer-reviewed.

## Declaration of competing interest

The authors declare that they have conflicts of interest.

## References

[1] Leape L.L., Brennan T.A., Laird N., Lawthers A.G., Localio A.R., Barnes B.A., Hebert L., Newhouse J.P., Weiler P.C., Hiatt H. (1991 Feb 7). The nature of adverse events in hospitalized patients: results of the Harvard Medical Practice Study II. N. Engl. J. Med..

[2] CRICO/RMF (2010). http://www.rmf.harvard.edu/high-risk-areas/diagnosis/index.aspx.

[3] Erdogan M., Ugurlu S., Ozdogan H., Seyahi E. (2019 Oct 29). Familial Mediterranean fever: misdiagnosis and diagnostic delay in Turkey. Clin. Exp. Rheumatol..

[4] Behar J., Corazziari E., Guelrud M., Hogan W., Sherman S., Toouli J. (2006 Apr 1). Functional gallbladder and sphincter of oddi disorders. Gastroenterology.

[5] Hyun J.J., Kozarek R.A. (2018 Sep 1). Sphincter of Oddi dysfunction: sphincter of Oddi dysfunction or discordance? What is the state of the art in 2018?. Curr. Opin. Gastroenterol..

[6] George J., Baillie J. (2007 Aug 1). Biliary and gallbladder dyskinesia. Curr. Treat. Options Gastroenterol..

[7] Agha R.A., Franchi T., Sohrabi C., Mathew G. (2020). The SCARE 2020 guideline: updating consensus surgical CAse REport (SCARE) guidelines. Int. J. Surg..

[8] Afghani E., Lo S.K., Covington P.S., Cash B.D., Pandol S.J. (2017 Jan 30). Sphincter of Oddi function and risk factors for dysfunction. Frontiers in nutrition.

[9] Crittenden J.P., Dattilo J.B. (2020 May 17).

[10] Sherman S., Lehman G.A. (2001 Nov). Sphincter of Oddi dysfunction: diagnosis and treatment. Jop.

[11] Lehman G.A. (1991 Jul 1). Endoscopic sphincter of Oddi manometry: a clinical practice and research tool. Gastrointest. Endosc..

[12] Meshkinpour H., Mollot M. (1992 Feb 1). Sphincter of Oddi dysfunction and unexplained abdominal pain: clinical and manometric study. Dig. Dis. Sci..

[13] Freeman M.L., DiSario J.A., Nelson D.B., Fennerty M.B., Lee J.G., Bjorkman D.J., Overby C.S., Aas J., Ryan M.E., Bochna G.S., Shaw M.J. (2001 Oct 1). Risk factors for post-ERCP pancreatitis: a prospective, multicenter study. Gastrointest. Endosc..

[14] Hogan W.J., Geenen J.E. (1988 Sep). Biliary dyskinesia. Endoscopy.

[15] Kalaitzakis E., Ambrose T., Phillips-Hughes J., Collier J., Chapman R.W. (2010 Dec). Management of patients with biliary sphincter of Oddi disorder without sphincter of Oddi manometry. BMC Gastroenterol..

[16] Kuncl R.W., Duncan G., Watson D., Alderson K., Rogawski M.A., Peper M. (1987 Jun 18). Colchicine myopathy and neuropathy. N. Engl. J. Med..

